# Translating Medicaid policy into practice: policy implementation strategies from three US states’ experiences enhancing substance use disorder treatment

**DOI:** 10.1186/s13012-021-01182-4

**Published:** 2022-01-06

**Authors:** Erika L. Crable, Allyn Benintendi, David K. Jones, Alexander Y. Walley, Jacqueline Milton Hicks, Mari-Lynn Drainoni

**Affiliations:** 1 Child and Adolescent Services Research Center, Department of Psychiatry, University of California, San Diego, 9500 Gilman Drive, La Jolla, San Diego, CA 92093 USA; 2 UC San Diego Dissemination and Implementation Science Center, La Jolla, San Diego, CA USA; 3 Clinical Addiction Research and Education (CARE) Unit, Section of General Internal Medicine, Department of Medicine, Boston Medical Center and Boston University School of Medicine, Boston, MA USA; 4 Department of Health Law, Policy & Management, Boston University School of Public Health, Boston, MA USA; 5 Department of Biostatistics, Boston University School of Public Health, Boston, MA USA; 6 Section of Infectious Diseases, Department of Medicine, Boston University School of Medicine, Boston, MA USA

**Keywords:** Policy implementation, Implementation science, Health policy, Public policy, Substance use disorder, Medicaid, Qualitative

## Abstract

**Background:**

Despite the important upstream impact policy has on population health outcomes, few studies in implementation science in health have examined implementation processes and strategies used to translate state and federal policies into accessible services in the community. This study examines the policy implementation strategies and experiences of Medicaid programs in three US states that responded to a federal prompt to improve access to evidence-based practice (EBP) substance use disorder (SUD) treatment.

**Methods:**

Three US state Medicaid programs implementing American Society of Addiction Medicine (ASAM) Criteria-driven SUD services under Section 1115 waiver authority were used as cases. We conducted 44 semi-structured interviews with Medicaid staff, providers and health systems partners in California, Virginia, and West Virginia. Interviews were triangulated with document review of state readiness and implementation plans. The Exploration, Preparation, Implementation, Sustainment Framework (EPIS) guided qualitative theme analysis. The Expert Recommendations for Implementing Change and Specify It criteria were used to create a taxonomy of policy implementation strategies used by policymakers to promote providers’ uptake of statewide EBP SUD care continuums.

**Results:**

Four themes describe states’ experiences and outcomes implementing a complex EBP SUD treatment policy directive: (1) Medicaid agencies adapted their inner/outer contexts to align with EBPs and adapted EBPs to fit their local context; (2) enhanced financial reimbursement arrangements were inadequate bridging factors to achieve statewide adoption of new SUD services; (3) despite trainings, service providers and managed care organizations demonstrated poor fidelity to the ASAM Criteria; and (4) successful policy adoption at the state level did not guarantee service providers’ uptake of EBPs. States used 29 implementation strategies to implement EBP SUD care continuums. Implementation strategies were used in the Exploration (*n*=6), Preparation (*n*=10), Implementation (*n*=19), and Sustainment (*n*=6) phases, and primarily focused on developing stakeholder interrelationships, evaluative and iterative approaches, and financing.

**Conclusions:**

This study enhances our understanding of statewide policy implementation outcomes in low-resource, public healthcare settings. Themes highlight the need for additional pre-implementation and sustainment focused implementation strategies. The taxonomy of detailed policy implementation strategies employed by policymakers across states should be tested in future policy implementation research.

**Supplementary Information:**

The online version contains supplementary material available at 10.1186/s13012-021-01182-4.

Contributions to the literature

- This study describes a taxonomy of policymaker-developed implementation strategies used to align state policy and provider practice.
- This study enhances our understanding of US federal, state, and organizational context determinants that impact EBP uptake in low-resource, public healthcare settings.
- This study demonstrates how the Exploration, Preparation, Implementation, Sustainment Framework, traditionally used to study organizational-level implementation, can be operationalized to examine federal and state policy implementation processes.
- This study demonstrates how qualitative methods can be used to describe and document policy implementation outcomes.

## Background

Policy researchers have widely studied health policy development and diffusion—the process by which decisions about whether to adopt a policy innovation are spread across governing bodies [1, 2]. Since the 1970s, public administration researchers have enhanced our understanding of how government agencies implement policies and measure implementation success or failure [3]. The emergent area of “health policy implementation science” is employing interdisciplinary approaches to examine variables and strategies that facilitate the adoption of evidence-based practices (EBPs) into clinical and community settings to align policy with practice [4]. This subfield is necessary to advance our understanding of how outer context factors mediate or moderate implementation efforts, including how organizations/agencies (comprised of policymakers, providers and other intermediaries) influence policy implementation processes and the impact of policy on population health outcomes [3, 5]. Despite the important upstream impact policy has on population health, few policy studies in health implementation science have examined implementation processes and strategies used to translate “Big P policies” from state and federal entities into accessible services in the community [6–9].

In 2015, the US Centers for Medicare and Medicaid Services (CMS; federal agency that oversees state-run Medicaid agencies) invited state Medicaid agencies to revise their benefits and adopt EBP care continuums for substance use disorder (SUD) treatment [10]. Medicaid agencies operate in each US state and territory to provide health insurance to low-income adults, children, and families. Medicaid agencies have historically provided limited coverage of substance use disorder (SUD) treatment services [11] and federal regulation prohibits them from paying for residential inpatient care for all beneficiaries [12]. The 2015 invitation from CMS allowed Medicaid agencies to waive federal restrictions and test new ways of administering Medicaid SUD benefits using Section 1115 waiver authority under the federal Social Security Act [13]. The “SUD waiver” opportunity encouraged Medicaid agencies to adopt care continuums including early intervention, outpatient, residential, and recovery support services. CMS recommended, but did not require, states to use the American Society of Addiction Medicine (ASAM) Criteria as a “nationally accepted” EBP care model to structure their care continuums [10]. Details on CMS and states’ selection of ASAM-aligned benefit arrays has been published elsewhere [14]. Broadly, the ASAM Criteria are proprietary guidelines for patient assessment, clinical services, discharge, and care transitions for individuals living with SUDs. Use of ASAM assessment criteria is associated with increased treatment retention [15], but more research is needed given widespread low fidelity to these criteria [16]. ASAM Criteria also describe a continuum of services including early intervention, outpatient, partial hospitalization, residential and intensive inpatient care [17]. For many states, implementing an ASAM Criteria-compliant SUD care continuum required a complete overhaul of their existing SUD treatment benefit structure, and new contracts with providers who had never participated in Medicaid.

State Medicaid agencies that adopted care continuums under the SUD waiver served as cases to identify policy implementation strategies used to adopt complex EBP care models across highly politicized [18], low-resource [19, 20] US health settings. Research examining the nature of implementation strategies as they are used in practice is critical since actual implementation activities often deviate from plans [21–23]. Investigating Medicaid SUD policy implementation reveals insights about multilevel factors that influence the translation of policy into accessible, lifesaving health services. Qualitatively describing implementation strategies and outcomes provides a contextual understanding of state-reported access and quality metrics associated with policy changes.

## Methods

Using three Medicaid SUD waiver demonstrations as cases, this study aimed to (1) describe common experiences and outcomes related to implementing national EBPs within state Medicaid contexts and (2) create a taxonomy of implementation strategies used by policymakers to promote providers’ uptake and delivery of statewide EBP SUD care continuums.

### Theory

**Fig. 1:**
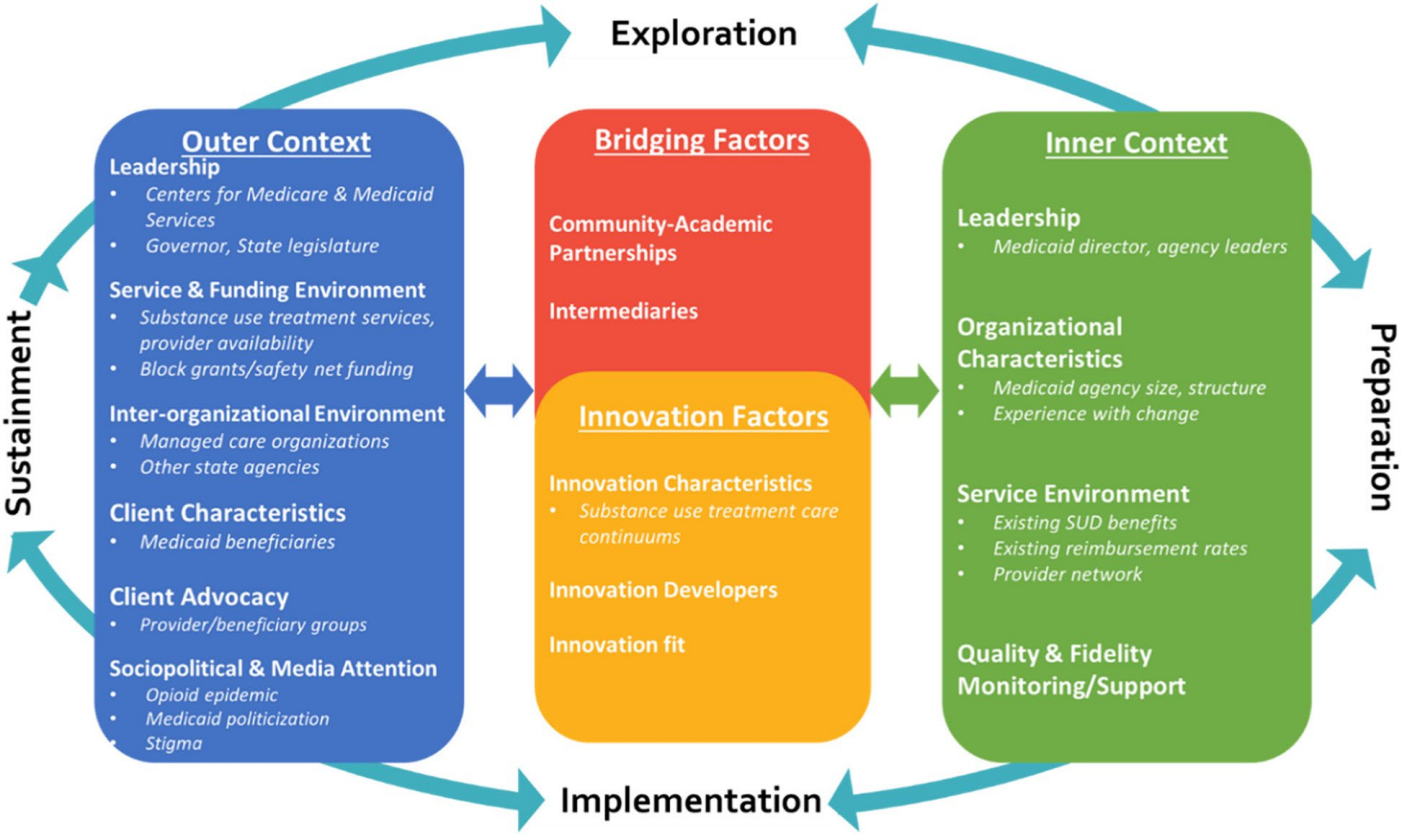
Adapted Exploration, Preparation, Implementation, Sustainment Framework for Medicaid agencies’ expansion of substance use treatment services

The Exploration, Preparation, Implementation and Sustainment Framework (EPIS) guided this investigation of states’ experiences implementing SUD care continuums. EPIS suggests that implementation unfolds over four phases. Stakeholders *explore* the need for change, *prepare* to adopt EBPs, and *implement* and *sustain* EBPs over time [24]. We adapted EPIS to examine Medicaid health policy implementation at the state-level (Fig. 1). In the *Exploration* phase, stakeholders become aware that the existing SUD treatment environment is inadequate to meet beneficiary needs. The decision to adopt a SUD care continuum (*innovation factors*) propels states into *Preparation* when stakeholders select EBPs for their continuums and plan for implementation. *Implementation* begins once states have CMS’s approval to initiate EBP use by Medicaid providers across the state. The *Sustainment* phase describes how states maintain their SUD care continuums over time.

Within each EPIS phase, inner and outer contextual factors influence implementation decisions and processes. The *inner context* describes the state Medicaid agency’s organizational characteristics and service environment (e.g., benefits, provider contracts). The *outer context* describes federal and state level influences, including how CMS and state leadership may impact state Medicaid benefit design. Given the politicization of Medicaid and SUD treatment [18, 25], “sociopolitical environment and media attention” were included as additional influences. The outer service environment includes all non-Medicaid SUD services such as care financed by federal and state safety-net funds, and providers that treat cash-only or privately insured populations. The interorganizational environment and network describes relationships between state health agencies, Medicaid managed care organizations (MCOs) who administer benefits, and non-governmental organizations (NGO) who often consult on Medicaid agency decisions [14]. *Innovation factors* represent the SUD care continuums that Medicaid agencies aimed to implement. The extent to which the SUD care continuum is aligned with the *inner* and *outer contexts* is highlighted in “innovation fit.” *Bridging factors* acknowledge that intermediary stakeholders or contractual arrangements are often necessary to span *inner* and *outer contexts* to improve innovation fit.

### Design

This research employed a multiple case study design in which each state was considered a case [26, 27]. This facilitated identifying common experiences across state Medicaid programs’ as they aimed to adopt SUD care continuums and employ policy implementation strategies.

### Study setting and sample

California, Virginia, and West Virginia Medicaid programs were selected as case studies. All three states were early adopters of the SUD waiver and had completed Exploration, Preparation and Implementation phases at the time of data collection. Comparing experiences across states at the same phase allows for a better understanding of how factors from the inner and outer contexts influenced their implementation processes. CMS approved California’s demonstration in 2015 [28], making it the first state to implement an EBP SUD care continuum, and a model to other states. Virginia’s and West Virginia’s demonstrations were implemented in 2017 [29] and 2018 [30], respectively.

Case study states were also selected for study inclusion due to the comprehensiveness of SUD benefit reform, and contrast in partisan landscapes and organizational characteristics. All three states planned to adopt care continuums that included outpatient, intensive outpatient, partial hospitalization, residential services, withdrawal management, medications for opioid use disorder (MOUD), and peer support services [31–33]. At the time of their SUD waivers, California and West Virginia each had politically aligned outer context leadership as indicated by their state Governors and the majority of state legislators being affiliated with the Democratic party. Virginia had not yet adopted Medicaid expansion [34] and had a politically divided Democratic Governor and majority Republican state legislature. At the time of implementation, Medicaid benefits covered nearly 26% of Californians, 12% of Virginians and 28% of West Virginians [35]. California has 58 county welfare departments that are responsible for implementing Medicaid benefits, while West Virginia and Virginia both administer benefits at the state level. The mix of commonalities and differences across these states made them ideal cases to compare and identify potentially transferable lessons that will inform other states’ future implementation efforts. A sample size of three US states as case studies was deemed sufficient for this research because the goal was to generate rich, detailed descriptions of each states’ context and implementation processes, which would not be feasible with a larger sample of states [36]. More details about case selection are reported elsewhere [14].

### Data collection

Semi-structured interviews were conducted with key informants from California, Virginia, and West Virginia state Medicaid programs and CMS. Key informants were initially identified by reviewing SUD waiver related policy documents for each agency and included individuals who served as innovation developers, leading the conceptualization and implementation of SUD care continuums. Snowball sampling techniques [36] were used to identify additional key informants in other state agencies, MCOs, providers, NGOs, and consultants involved in states’ implementation processes.

The interview guide included questions about influential factors identified in EPIS. Participants were asked to describe *inner context* features such as the state Medicaid program’s organizational structure, leadership, existing SUD benefits, and provider network. Additional topics included *outer context* influences including state and federal leadership, the nature of the state SUD service environment, and *bridging factors* used to enhance statewide adoption of the SUD care continuum.

Key informants were interviewed between July 2019 and April 2020. Interviews lasted approximately 1 h, were audio-recorded, and were professionally transcribed. Data collection continued until the research team agreed that thematic saturation was achieved for each state, and additional data collection would not yield new insights [37, 38]. Publicly available Medicaid demonstration concept papers, applications, approval letters, readiness review documents detailing service gaps, and implementation plans were sourced from state agency websites for document review.

### Data analysis

An initial qualitative codebook was developed with codes based on constructs described in EPIS [24] and definitions from Moullin et al. [39]. Code definitions and inclusion criteria were modified to align with model adaptations. Transcripts were coded so that each relevant passage was assigned to an EPIS construct and phase. For example, a passage describing the pre-implementation Medicaid SUD provider network as understaffed was coded to *inner context* codes and *exploration* because stakeholders described an existing deficiency in Medicaid’s provider network. Any transcript or document passages describing an implementation activity were coded as “implementation strategy” and an appropriate EPIS-related code to facilitate identification in during thematic analysis.

Two graduate-level trained qualitative researchers double-coded 25% of the interviews to ensure coding consensus and resolved discrepancies through consensus discussions. Both researchers were knowledgeable about Medicaid policy and SUD treatment. The lead researcher also has prior experience working with CMS and state Medicaid agencies. Once overall coding consensus was achieved and no new edits were needed to clarify codebook definitions, the lead researcher coded the remaining transcripts and all documents. Documents were primarily coded to identify and describe implementation strategies. We performed a directed content analysis [40] by reviewing and summarizing transcript and document data coded to each construct and EPIS phase to identify themes. The research team discussed and reached consensus on final themes and implementation strategies. All coding and analysis were conducted in NVivo 12.0 [41].

Implementation strategies described in interviews and document review were cross-walked to a list of 73 standardized strategy names and definitions developed by the Expert Recommendations for Implementing Change (ERIC) project. Original ERIC strategies were derived from Delphi processes with implementation science experts [42]. Recent modifications to ERIC strategies were also considered [23]. ERIC strategy cluster names [43] (e.g., financial strategies) were used to thematically group implementation strategies by action and intent. Each implementation strategy was described using the Proctor et al. Specify It criteria [44] such that each strategy was labeled with an ERIC name, actors, actions, targets, temporality, implementation outcome, and justification [44]. “Temporality” was specified as the EPIS phase during which each implementation strategy was employed.

### Ethics review and reporting standards

This study was determined to be exempt by the Boston University Medical Campus Institutional Review Board (H-38990). Qualitative data are reported in compliance with the Standards for Reporting Qualitative Research recommendations [45] (Appendix 1).

## Results

### Participant characteristics

Thematic saturation was achieved after conducting 44 stakeholder interviews across states (CA, *n*=17; VA, *n*=16; WV, *n*=9) and CMS (*n*=2). Across states, interviews were conducted with six categories of key informants: CMS staff (*n*=2), Medicaid staff (*n*=17), other state agency staff (*n*=9), NGOs and external consultants (*n*=6) that supported Medicaid agencies’ implementation efforts, providers (*n*=6), and MCO representatives (*n*=4) involved in the delivery of new services. Key stakeholder titles and position descriptions were omitted to preserve confidentiality. Fifteen documents were included in qualitative analysis including concept papers, applications, and implementation plans from each state.

### Implementation themes

Qualitative analysis revealed four themes describing states’ policy implementation outcomes: (1) Medicaid agencies adapted their inner/outer contexts to align with EBPs and adapted EBPs to fit their local context; (2) enhanced financial reimbursement arrangements were inadequate bridging factors to achieve statewide adoption of new SUD services; (3) despite trainings, service providers and MCOs demonstrated poor fidelity to the ASAM Criteria; and (4) successful policy adoption at the state level did not guarantee service providers’ uptake of EBPs. The first three themes reveal policy implementation outcomes (i.e., context adaptation, policy and service adoption, fidelity) that occurred due to innovation fit issues throughout Implementation. The final theme describes how poor service adoption represents a threat to Sustainment.

### Implementation adaptation: Medicaid agencies adapted their inner/outer contexts to align with EBPs and adapted EBPs to fit their local context

CMS permitted states to choose any “industry standard models” [10] to structure their SUD care continuums. Although no state reported uniform use of ASAM Criteria for patient assessment or clinical care requirements prior to implementation, all three early adopters chose to model their new care continuums on ASAM Criteria. This decision produced significant innovation fit issues between the inner and outer contexts. Throughout the Implementation phase, key informants described a tension between eagerness to implement ASAM as a “national standard of care” and knowing that doing so require “a pretty broad spectrum of change across the system in order to make this happen.”

To address this innovation fit challenge, Medicaid agency staff considered ways to modify their outer service environments to comply with ASAM Criteria. All states cross-walked state provider licensing regulations to ASAM levels to determine which providers qualified to deliver new SUD services. Key informants considered altering state regulations to conform with ASAM Criteria to promote EBP fidelity and standardize SUD treatment across Medicaid and non-Medicaid providers. Ultimately, only one state modified statewide regulations and required all providers to obtain an “ASAM designation” from the state indicating they were providing care consistent with ASAM Criteria. As one California Medicaid agency representative said, “we created an ASAM designation… we’re doing it for all of our licensed facilities, regardless of the funding source.”

Key informants in Virginia and West Virginia were hesitant to adapt their local settings to meet ASAM Criteria because they wanted to remain open to evolving science in SUD treatment and new EBPs. A Virginia state agency representative said, “if next year someone comes out that says that there's another better evidence-based way of testing for substance abuse treatment and level of care, then you would have to change the regulations again to look like that better mode of assessing.”

All three Medicaid programs edited provider contracts, manuals, prior authorization, and medical necessity forms to include language specific to ASAM Criteria thereby using these documents as *bridging factors* to promote ASAM Criteria adoption. Medicaid staff developed policy manuals that “followed along the criteria for different levels of care so that it flows with the ASAM Criteria.” Medicaid MCO provider contracts stipulated that providers receive an ASAM designation indicating which level of care they were qualified to provide. Provider compliance with ASAM designation was verified at on-site evaluations by Medicaid staff in California and by external vendors in Virginia and West Virginia. A Virginia Medicaid agency representative described this process as “a way to validate all the programs as ASAM.” During these visits, providers shared “their treatment protocols, their staffing, their service capacities, collateral affiliations and all that. [The contractor] would assess all those structural components and deem them an ASAM provider.”

Across states, Medicaid staff and MCO key informants said they initially wanted to achieve fidelity to ASAM Criteria, but ultimately adapted the model to improve the fit between ASAM Criteria and inner/outer service environments. For example, ASAM Criteria include requirements for the number of clinical service hours provided in each level of care. California providers felt requirements were overly burdensome. A California Medicaid agency representative recalled, “our providers said it's just too much, it's too overwhelming.” Rather than forcing providers to adapt their service delivery practices, California Medicaid modified their benefit requirements, replacing some of the clinical service hours with less intensive therapeutic service requirements.

Virginia Medicaid reduced provider documentation requirements for patients’ biopsychosocial needs assessments. A Virginia Medicaid MCO representative described the ASAM documentation requirements as “pretty time consuming.” Providers “were not used to doing that, and with their volume, [they] had no interest in doing that.” As a result, Medicaid MCOs worked with providers to determine “what was the minimum amount of information we needed to be able to make a reasonable adjustment – a reasonable decision on ASAM… They didn't have to fill out the form in its excruciating entirety to get in. They had to give us enough that we could make the judgement if they met criteria. So, we did cut some corners on that.” States initially planned to modify inner and outer service environments to meet the ASAM Criteria. However, their desire to remain open to new EBPs and provider fit issues ultimately forced them to modify the EBP to meet local setting needs.

### Implementation outcome: enhanced financial reimbursement arrangements were inadequate bridging factors to achieve statewide adoption of new SUD services

Across states, costs associated with delivering new SUD services negatively impacted providers working in Medicaid service environments. Providers experienced financial hardship in the Implementation phase when (1) they enhanced service delivery models to meet new staffing and clinical requirements with unknown beneficiary demands and (2) when reimbursement payments were delayed.

Key informants in California’s county-run Medicaid programs were reluctant to implement new SUD services out of fear that the start-up costs and demand for services would be financially infeasible with their existing organizational resources. As one California NGO partner noted, “there has been a lot of sort of fiscal and budget uncertainty that counties have had to deal with because… these were services that hadn’t been offered through Medi-Cal before.” Financial uncertainty resulted in rural counties with smaller operating budgets choosing to not adopt SUD EBPs.

Across states, Medicaid providers worried that the costs associated with new administrative processes and new services such as MOUD and case management that were not part of their pre-demonstration delivery model, would be substantial. As one West Virginia behavioral health agency representative said, “There were certainly all kinds of questions from the providers that nobody could answer at the time…Mostly it's just about financial stability.” Eventually these pre-implementation fears were actualized. One provider said that without having a “financial war chest,” and being a larger organization with “a diverse array of services, we would never have been able to do this.” The provider noted that “a small provider would die trying to do this… to invest what was gonna be needed to play ball in the new system. They were just never gonna get there.”

Across states, providers described routinely being denied or receiving delayed reimbursements due to new challenges with new documentation and billing systems. A California provider noted that, “they’re still trying to fix what’s broken” and said the Medicaid agency “had to work out making interim payments to the providers outside the whole [electronic health record] because they knew damn well that the provider’s going to go out of business if they can’t get their revenue to meet their payroll needs… It’s been a tremendous mess.” In Virginia and West Virginia, providers said they used different reimbursement forms for each Medicaid MCO. Inconsistent billing processes and providers’ limited familiarity with new documentation requirements resulted in significant unpaid claims and “a little bit of chaos that we’re having to work through.” One Virginia provider said that MCOs owed “so much back money” to the practice, that the provider was contemplating denying services to beneficiaries of certain Medicaid MCOs… “We might put a sign up on the front door and say, ‘Members of [MCO] are no longer welcome to receive services here’… and just hold that out”

Medicaid agencies anticipated monetary risks associated with implementing new SUD services and implemented financial reimbursement arrangements as bridging factors to support providers. California allowed providers to claim reimbursement for time spent documenting patient biopsychosocial needs and care plans, and some counties funded providers’ administrative or new service start-up costs. West Virginia allowed providers to continue receiving reimbursements from state safety net funds while they experimented with billing documentation for new services. A West Virginia behavioral health agency representative said this was done “so providers wouldn’t be afraid to try” to provide and bill for new services. All three Medicaid programs increased reimbursement rates to entice providers to deliver new Medicaid services. However, these financial bridging factors were insufficient to offset the substantial costs experienced by service providers who adopted policy-directed SUD EBPs.

### Implementation outcome: despite trainings, service providers and MCOs demonstrated poor fidelity to the ASAM Criteria

Key informants across states said providers and MCOs did not achieve fidelity to the ASAM Criteria, revealing a critical Implementation phase policy outcome, and fit issues between the EBP model and existing inner/outer service environments. Prior to states’ demonstrations, providers in California, Virginia, and West Virginia had limited experience using ASAM level requirements and the model’s biopsychosocial assessment. Key informants said that implementing ASAM Criteria required “a heavy logistical lift” as providers gained new clinical knowledge. Rather than quantitatively measuring fidelity to ASAM Criteria, stakeholders said they generally assessed how well service providers adopted a “cultural understanding about substance use disorder as a chronic illness” characterized by cycles of relapse and recovery.

Key informants said all three Medicaid programs invested heavily in webinars and in-person workshops to train providers and healthcare administrators on technical care delivery and documentation requirements. Across states, key informants said, “a lot of providers struggled tremendously to apply the ASAM Criteria.” One Virginia MCO representative said the criteria were especially challenging to apply in “a system that was used to really almost no criteria, to be honest.” Despite trainings, stakeholders said, “it's still a struggle and that there's a lot of providers who still don't a hundred percent get the criteria.” A CMS representative said that “a lot of providers, you know, might say that they use it fair, but when you look at how they do assessments and or when you look at the programming that they deliver, there's a lot of daylight between that and the textbook that ASAM published.” States invested time and money to educate providers about the ASAM Criteria with the goal of promoting fidelity.

Fidelity to ASAM Criteria was also reduced when Medicaid MCOs’ denied claims or required prior authorizations for services that were covered under the new Medicaid benefits. For example, Virginia providers complained that Medicaid MCOs unnecessarily required prior authorizations or documentation that beneficiaries were abstaining from substance use in order to prescribe MOUD. As one Virginia provider said, “if you have somebody who had a urine drug test as positive for cocaine, you would have to get on the phone with the insurance company and explain to them why being positive for cocaine didn’t mean that they shouldn’t get their buprenorphine.” Such MCO requirements were considered “non-evidence grounded” barriers to treatment that prevented model fidelity.

Beyond clinical service requirements, key informants said some providers and Medicaid MCOs struggled to embrace the ASAM Criteria’s biopsychosocial patient assessment. Providers and MCO staff struggled with “shifting their mindset from…everybody starts at this level of care then goes to the next level of care” to a model where service selection is based on individual client needs. Providers and MCOs that did not view SUD as a chronic illness lacked fidelity to ASAM-oriented care when they ended treatment or denied care to clients who relapsed to drug use. A West Virginia Medicaid agency representative said the state often had to negotiate denied claims with MCOs who did not understand that “people are going to fail and come back” due to the relapsing and remitting nature of substance use.

Across states, Medicaid staff said they are continuing to offer provider trainings to improve fidelity to ASAM Criteria. West Virginia is planning to purchase a software (i.e., CONTINUUM) developed by the publishers of the ASAM Criteria for Medicaid providers with the goal of improving fidelity to the biopsychosocial patient assessment. Virginia Medicaid considered asking providers and MCOs to use the CONTINUUM software. However, MCOs pushed back citing, “you had to license it… We weren’t going to defer our utilization management to some outside tool…that we would have to follow regardless if we agreed with it or not.” Key informants highlighted tensions between provider and MCO autonomy and Medicaid’s desire for ASAM fidelity.

### Sustainment outcome: successful policy adoption at the state level did not guarantee service providers’ uptake of EBPs

Early adopter states used 1115 waiver authority to successfully adopt Medicaid policies that established reimbursement for additional EBPs for SUD treatment. However, achieving provider adoption of these new Medicaid SUD services requires “a continuous quality improvement process.” States entered the policy Sustainment phase but are revisiting Implementation phase activities to address limited EBP adoption of recovery support services and to address provider workforce shortages.

Service providers have “struggled with billing care coordination or case management” which has prevented uptake of recovery support services. In California, Medicaid agency staff noted, “we're doing a bunch of it through grant funding right now, but not through the Medicaid system.” Post-implementation, states continue to rely on state block grant dollars (federal funds allocated to states for behavioral health services) and other state funds rather than just Medicaid reimbursement to pay for treatment.

Inadequate provider networks were identified by key informants as a major outer context threat to maintaining newly implemented SUD care continuums, especially in rural areas of states. One California NGO partner described how counties are “concern[ed] about essentially committing to deliver services that you may not be able to find the providers to deliver.” Key informants across states said the demand for services, especially residential treatment and MOUD, outpaced existing provider capacity, resulting in treatment waitlists.

A lack of licensed providers in the outer context service environment limited Medicaid and MCOs’ ability to contract providers to adopt these services during Preparation and Implementation phases. Key informants said there were not enough providers with expertise needed to treat individuals with multiple chronic comorbidities. A California Medicaid agency representative noted that “the referrals we get are very high co-occurring SMI [serious mental illness], comorbid, tri-morbid” and that “because of the exponential growth” in substance use, the existing workforce is “just not enough.” A Virginia provider said the workforce shortage “has been a crisis for what, four or five years now in America, and it’s gotten worse instead of better. You can’t find a psychiatrist that has any interest at all working in community behavioral health hardly.” Medicaid agencies and MCOs fear that without more qualified providers, new SUD care continuums will not be sustained.

#### Taxonomy of implementation strategies used across states’ health policy implementation efforts

States used 29 implementation strategies to implement SUD care continuums. Implementation strategies were used in the Exploration (*n*=6 strategies used), Preparation (*n*=10), Implementation (*n*=19), and Sustainment (*n*=6) phases and primarily focused on developing stakeholder interrelationships (*n*=11), evaluative and iterative approaches (*n*=7), financing (*n*=6), training and educating new stakeholders (*n*=5), and providing interactive assistance (*n*=5) (Table 1). Additional details of Table 1 strategies are described in the subsequent sections by EPIS phase. Appendix 2 details each implementation strategy by ERIC name, temporality, actors, actions, targets, dose, implementation outcome, justification, and state(s) where the strategies were used.

**Table 1:** Policymaker implementation strategies by expert recommendations for implementing change cluster and Implementation phase

Implementation strategy cluster	Exploration (***n***=6 strategies)	Preparation (***n***=10 strategies)	Implementation (***n***=19 strategies)	Sustainment (***n***=6 strategies)
Develop stakeholder interrelationships (*n*=11 strategies)	• Promote network weaving • Use advisory boards and workgroups (*n*=2)^a^	• Promote network weaving • “Visit” other sites • Identify and prepare champions	• Promote network weaving • Identify early adopters • Capture and share local knowledge • Build a coalition	• Work with educational institutions
Utilize financial strategies (*n*=9 strategies)	• N/A	• Fund and contract and/or negotiate with vendors for the clinical innovation • Access new funding	• Alter incentive/ allowance structures (*n*=3)^a^ • Place innovation on FFS/formularies • Make billing easier	• Alter patient/ consumer fees • Alter incentive/ allowance structures
Use evaluative and iterative strategies (*n*=7 strategies)	• Conduct local needs assessment	• Assess for readiness and identify barriers and facilitators • Develop a formal implementation blueprint • Obtain and use patient/consumer and family feedback	• Stage implementation scale up	• Develop and organize quality monitoring systems • Purposively reexamine the implementation
Train and educate stakeholders (*n*=5 strategies)	• N/A	• N/A	• Conduct educational meetings • Develop educational materials (*n*=2)^a^ • Conduct educational outreach visits • Distribute educational materials	• N/A
Provide interactive assistance (*n*=5 strategies)	• Centralize technical assistance	• Centralize technical assistance	• Provide local technical assistance • Centralize technical assistance (*n* = 2)^a^	• N/A
Change infrastructure (*n*=3 strategies)	• Change credentialing and/or licensing standards	• Crosswalk EBP/innovation with existing service environment *(new)*	• Change credentialing and/or licensing standards	• N/A
Adapt and tailor to context (*n*=1 strategies)	• N/A	• N/A	• N/A	• Use data experts
Support clinicians (*n*=1 strategies)	• N/A	• N/A	• Develop resource sharing agreements	• N/A

*Notes*: ^a^All strategies were used by case study state(s) once per phase unless otherwise indicated (e.g., *n*=2 indicates that a given strategy was used twice during the indicated phase phase)

Implementation strategy names clusters were identified from the Expert Recommendations for Implementing Change project

### Exploration

CMS leadership and state agencies *used advisory boards and workgroups* and *conducted local needs assessments* to establish consensus on the need to improve Medicaid SUD benefits. CMS leadership *promoted network weaving* to develop positive, interorganizational working relationships with the publishers of ASAM Criteria prior to recommending the model to states. CMS also *centralized technical assistance* to promote consistent information about demonstration requirements.

### Preparation

Preparation featured three strategies focused on developing stakeholder interrelationships. Medicaid agency staff *promoted network weaving* and conducted remote *visits with other sites* to identify EBPs and implementation lessons. Virginia *identified and prepared champions* throughout state agencies to obtain widespread buy-in. All states used financial strategies (*n*=2) to *access new funding* to increase Medicaid budgets, and *fund and contract* with new providers. Evaluative and iterative strategies (*n*=3) were critical to developing SUD care continuums benefits. CMS required that states *assess for readiness and identify barriers and facilitators* and *develop a formal implementation blueprint* prior to receiving demonstration approval. Medicaid agencies conducted public comment periods to *obtain and use patient/consumer and family feedback* about the planned innovation. States used one non-ERIC implementation strategy related to changing infrastructure; states *cross-walked the EBP/innovation with existing service environment* to match existing state licensing standards to ASAM Criteria.

### Implementation

States used more strategies during Implementation (*n*=19) than any other phase. States continued to *develop stakeholder interrelationships* (*n*=4) by *promoting network weaving, capturing and sharing local knowledge* and *identifying early adopters* as models for implementation. States used financial strategies (*n*=3) to incentivize providers to deliver new services, trained and educated providers (*n*=5) about ASAM Criteria.

### Sustainment

All three Medicaid agencies are *working with educational institutions* to formally evaluate changes in SUD service access. Key informants actively employ evaluative and iterative strategies (*n*=2) such as *developing and organizing quality monitoring systems* to *purposively reexamine the implementation of new benefits* and identify gaps in their care continuums. In Virginia, financial strategies (*n*=2) are helping to sustain new benefits. Virginia legislature passed a law banning providers from charging Medicaid beneficiaries’ cash for MOUD (*altering patient/consumer fees)*, and the state behavioral health agency *altered incentive/allowance structures* to encourage more providers to become waivered buprenorphine prescribers.

## Discussion

We identified four themes that describe states’ outcomes implementing and sustaining complex EBP innovations: (1) Medicaid agencies adapted their inner/outer contexts to align with EBPs and adapted EBPs to fit their local context; (2) enhanced financial reimbursement arrangements were inadequate bridging factors to achieve statewide adoption of new SUD services; (3) despite trainings, service providers, and MCOs demonstrated poor fidelity to ASAM Criteria; and (4) successful policy adoption at the state level did not guarantee service providers’ uptake of EBPs. Although US states’ 5-year 1115 SUD waiver demonstrations are ongoing, our qualitative themes help to contextualize state-reported mid-point quantitative measurements of service access and quality. California counties have reported wide variation, but an overall significant increase in the number of Medicaid beneficiaries receiving SUD services following waiver implementation [46]. Similarly, Virginia reported a 57% increase in Medicaid beneficiaries’ use of SUD services [47]. West Virginia’s mid-point evaluation data were not available at the time of publication, but the state has reported an increase in referrals for residential treatment [48]. Overall, state Medicaid agencies successfully adopted new policies and, according to their evaluations, have generally increased beneficiaries’ access to care. But our study illuminates the specific implementation strategies and descriptive outcomes from translating policy into practice.

Few studies have used implementation frameworks to examine policy implementation processes, strategies and outcomes [6]. This study demonstrates how implementation frameworks like EPIS can be adapted to explain outer and inner context determinants that impact policy implementation processes and outcomes. Our examination of strategies within the EPIS Framework enhances our knowledge of how contracts can serve as bridging factors to support implementation across contexts [49]. Medicaid agencies are one of many SUD treatment payers, but nonetheless they were able to use Medicaid contracts to wield influence beyond their inner context service environment. However, agency influence and policy adoption were insufficient to achieve provider EBP adoption and fidelity. While providers complied with Medicaid agency contracts to become ASAM designated, they still lacked fidelity to ASAM Criteria service requirements. The bi-directional nature of *inner* and *outer context* influences was demonstrated by providers’ ability to convince Medicaid agencies to relax burdensome ASAM Criteria requirements, thereby fitting the EBP to their outer context needs.

This study also suggests that some bridging factors may not be strong enough to achieve desired implementation outcomes. Although Medicaid agencies reimbursed providers for time spent documenting care and used grant funds to support providers’ transition to new billing systems, providers were still financially exposed which prevented some from adopting new SUD services. Case study states used financial reimbursement strategies that were inadequate bridging factors to promote provider adoption of EBPs. Billing issues, administrative burdens, and delayed reimbursement are commonly cited reasons why providers do not participate in Medicaid [20]. Thus, increasing reimbursement rates alone may be insufficient to convince providers to contract with Medicaid. Implementation strategies and bridging factors that effectively reduce administrative burdens and facilitate timely reimbursement for services are needed to improve provider participation [50] and implementation of new services.

Limited fidelity to the ASAM Criteria highlights additional innovation fit issues. EPIS suggests that EBP innovation fit is a product of the inner and outer contexts, and a requirement for implementation success [24, 39]. The published ASAM Criteria provide a detailed description of each service level, but they do not provide fidelity measurement tools. Additional services and tools (e.g., Commission on Accreditation of Rehabilitation Facilities ASAM Level of Care Certification program, CONTINUUM software, ASAM Criteria Training Courses) must be purchased to monitor fidelity. At the time of data collection, only West Virginia planned to purchase CONTINUUM software to promote provider fidelity to ASAM Criteria. Although Medicaid agencies and partners invested heavily in training providers on SUD innovations, the cultural and clinical shifts required of providers and MCOs to realize model fidelity was substantial and remains unachieved. Poor innovation fit also results in limited provider adoption of EBPs [51]. Misalignment between the planned EBPs and outer context workforce capacity prevented Medicaid providers from adopting recovery support services and sufficient residential treatment capacity. California’s 1115 SUD waiver midpoint evaluation confirms that recovery support service claims have been paid for less than 3% of Medicaid beneficiaries since the EBP was added to the benefit array [46]. Additionally, the behavioral health workforce shortage is a national problem and important constraint affecting states’ service environments and innovation capacity [52]. Our study findings emphasize the need to consider how policy initiatives fit with the broader outer context [6]. Our results provide detailed descriptions of policy-fit challenges, but the health policy implementation field would benefit from rigorous quantitative measures to identify outer context determinants that impact implementation success [6]. These measures should be assessed throughout Preparation, Implementation, and Sustainment phases to proactively address areas of resource constraints, political misalignment, and other potential determinants.

Cataloging the 29 implementation strategies used in states’ SUD waivers furthers methodological goals of identifying and specifying activities that effectively translate policy into clinical practice. Similar to prior research [53], we identified few implementation strategies used during states’ Exploration phase. The resource-intensive nature of Exploration strategies (e.g., workgroups) may explain why less were used. Like our study, prior large-scale systems implementation efforts also show a concentration of implementation strategy use during Preparation and Implementation phases [23, 53, 54]. Strategies used in the Implementation phase demonstrate the importance of stakeholder interrelationships and the sheer volume of training and education-based strategies required to introduce a system of service providers to a new standard of care. Limited use of implementation strategies in the Sustainment phase combined with documented care gaps suggests that states will need additional strategies to maintain SUD care continuums.

Finally, this study enhances our understanding of policy implementation strategies by providing a specified a taxonomy of ERIC implementation strategies used across all four EPIS phases to implement SUD waiver policies. This research builds on prior work demonstrating the generalizability of ERIC strategies in health policy implementation research [9, 23]. Purtle et al. categorized ERIC strategy use by state mental health agencies to promote federal behavioral health parity implementation [9]. Like Purtle et al., we found that providing interactive assistance was a frequently used policy implementation strategy during the Implementation phase. While Purtle et al. identified low levels of interagency collaboration, we found that state policymakers invested time and resources in developing stakeholder interrelationships throughout all EPIS phases. Research is needed to explore the utility of each strategy, or bundled strategies, for promoting adoption of evidence-based substance use treatment services. Our study and others [9, 23] suggest directions for future health policy implementation science including further identification and specification of strategies for promoting policy adoption, and quantitative measurements of the utility of health policy implementation strategies in global settings.

### Strengths and limitations

The breadth of stakeholder perspectives is a strength of this research. Interviewees’ recall bias may limit this research since many implementation strategies were used a year or more before the interview. However, most implementation strategies described in interviews were confirmed in document review. Themes and implementation strategies were identified across three states, suggesting transferability of findings, although implementation experiences may differ for states adopting later as outer context influences change. Including more US states as case studies could provide more insights into other state policy implementation experiences. Two of the states included in this study had Democratic state leadership (i.e., legislators and Governor) when the Medicaid agencies submitted their SUD waivers, which likely influenced the nature of new services included in the benefit and speed with which waiver policies were initially supported and implemented [14]. Prior research suggests that while both conservative and liberal states provide legislative support for substance use treatment related initiatives, Republican controlled states provide substantially less funding for these services compared to Democrat controlled states [55]. Study themes may have limited transferability to states with Republican Governors and legislative majorities that fund less comprehensive care continuums. Additional research on Republican controlled waiver states is needed. Finally, the researchers are US based and this study highlights implementation challenges in a public healthcare setting that is specific to the US context (i.e., Medicaid), which limits transferability to countries where healthcare delivery systems are organized differently.

## Conclusion

Three states’ experiences adopting SUD care continuums within their Medicaid benefit revealed four themes related to Implementation and Sustainment of EBPs in low-resource, public healthcare settings. States experienced similar policy implementation outcomes related to context adaptation, successful policy but limited service adoption, and limited fidelity to an EBP model of care. This study demonstrates how qualitative methods can be used to describe and document policy implementation outcomes. Future research should test the utility of each identified policy implementation strategy to enhance their transferability across policy innovation and inner and outer contexts.

## Supplementary Information

**Additional file 1: Appendix 1.** Standards for Reporting Qualitative Research Checklist

**Additional file 2: Appendix 2.** Implementation strategies used to enhance substance use disorder treatment across state Medicaid agencies

## Data Availability

The qualitative interview data analyzed for this study are not publicly available because they were generated in interviews with the research team, with the expectation that participant identity would be kept confidential. De-identified transcripts may be available from the corresponding author on reasonable request. Data analyzed in document review are from publicly available records.

## Abbreviations

ASAM: American Society of Addition Medicine
CMS: Centers for Medicare and Medicaid Services
EBP: Evidence-based practice
EPIS: Exploration, Preparation, Implementation, Sustainment Framework
ERIC: Expert Recommendations for Implementing Change
MCO: Managed care organization
MOUD: Medication for opioid use disorders
NGO: Non-governmental organization
SUD: Substance use disorder
