# Supplementary material for: Translating Medicaid policy into practice: policy implementation strategies from three US states’ experiences enhancing substance use disorder treatment
Source: Implement Sci. 2022 Jan 6;17:3. doi: 10.1186/s13012-021-01182-4 (PMC8734202; doi:10.1186/s13012-021-01182-4)
Supplement: Supplementary file 2 — Additional file 2: Appendix 2. Implementation strategies used to enhance substance use disorder treatment across state Medicaid agencies [file 13012_2021_1182_MOESM2_ESM.docx]

**Appendix 2. Implementation Strategies Used to Enhance Substance Use Disorder Treatment Across State Medicaid Agencies**

| **Implementation Strategy Name from Literature*** | **Number of States** | **Temporality**  **(EPIS Phase)** | **Actors** | **Actions** | **Action**  **Targets** | **Dose^+^** | **Implementation Outcome** | **Justification** | |
| --- | --- | --- | --- | --- | --- | --- | --- | --- | --- |
| Promote network weaving | N/A | Exploration | CMS leadership | Discuss model requirements  Discuss copyright concerns | The ASAM Criteria developers | Multiple conversations | Preemptively address any concerns associated with promoting use of ASAM Criteria  [Acceptability] | ASAM Criteria is a trademarked care model | |
| Use advisory boards and workgroups | N/A | Exploration | CMS leadership, U.S. Department of Health and Human Services leadership, representatives from state Medicaid agencies, SUD experts | Discuss EBPs models of care for SUD treatment  Establish consensus on national standard of care | State Medicaid programs | Multiple conversations | Recommend an evidence-based model to define SUD care continuums  [Acceptability, Appropriateness] | Lack of EBPs for SUD treatment  CMS wanted states to adopt full SUD care continuums | |
| Use advisory boards and workgroups  Conduct local needs assessment | 3 | Exploration | Governors,  State health and behavioral health departments,  Medicaid agencies,  non-governmental organizations | Discuss nature of Medicaid beneficiary SUD treatment needs  Discuss local impact of opioid epidemic | Medicaid SUD benefits  State SUD treatment needs | Multiple meetings over several months | Establish consensus on need for enhanced Medicaid SUD benefits  [Acceptability, Appropriateness] | High prevalence of substance misuse  Inadequate provider networks, lack of EBPs | |
| Change credentialing and/or licensing standards | 1 | Exploration | Medicaid agency leadership, CMS leadership | Re-assess residential facilities classified under IMD | Residential facilities | Multi-day tour of facilities | Allow Medicaid to reimburse residential treatment facilities  [Adoption] | Lack of Medicaid reimbursable residential treatment | |
| Centralize technical assistance | 3 | Exploration | CMS leadership | Education and outreach directly to Medicaid agencies | State Medicaid leadership | Continuous | Prepare a Section 1115 waiver to enhance SUD treatment  [Adoption] | Medicaid agencies varied in their familiarity with waiver demonstra-tions | |
| Promote network weaving | 3 | Preparation *(continued through Implement-ation)* | Medicaid agency innovation developers | Outreach to other state Medicaid programs, non-governmental organizations, consultants  Education on EBPs, strategies to implement EBPs | Medicaid benefit structure | Multiple calls, internet searchers | Identify EBPs to support SUD care continuum  [Appropriateness]Identify lessons learned from implementation efforts in other states  [Feasibility] | Medicaid agencies were unfamiliar with EBPs | |
| “Visit” other sites | 3 | Preparation | Medicaid agency innovation developers, state behavioral health department | Education on EBPs | Medicaid benefit structure | Multiple calls in place of in-person visits, internet searches | Identify EBPs to support SUD care continuum  [Appropriateness, Feasibility] | Medicaid agencies wanted to learn from others experiences | |
| Crosswalk EBP/innovation with existing service environment  **[New strategy]** | 3 | Preparation | Medicaid agency innovation developers, state behavioral health department | Direct comparison of state license regulations to ASAM Criteria | SUD treatment Providers, facilities | Undetermined | Promote fidelity to ASAM Criteria  [Fidelity] | Medicaid agencies, providers were unfamiliar with ASAM requirements | |
| Centralize technical assistance | 3 | Preparation | CMS leadership, Medicaid Innovation Accelerator Program consultants | Direct outreach to Medicaid agencies  Local support to design innovation | Medicaid agency leadership and innovation developers | Continuous | Receive CMS approval for 1115 waiver demonstration  [Adoption] | Medicaid agencies varied in their familiarity with 1115 waiver de-monstrations and ASAM Criteria | |
| Fund and contract and/or negotiate with vendors for the clinical innovation | 3 | Preparation | Medicaid MCOs | Provider recruitment  Education about new services, increased reimbursement rates | SUD treatment Providers | Continuous | Expand Medicaid SUD provider network  [Adoption] | Medicaid agencies needed to meet network adequacy requirements to gain demonstration approval | |
| Assess for readiness and identify barriers and facilitators | 3 | Preparation | Medicaid agency | Assess provider network, beneficiary needs | SUD treatment Providers, beneficiaries | Once | Complete readiness review assessment  [Appropriateness] | CMS required task | |
| Develop a formal implementation blueprint | 3 | Preparation | Medicaid agency | Identify strategies to implement new services | SUD treatment Providers, beneficiaries | Once | Develop implementation plan  [Feasibility, Adoption] | CMS required task | |
| Obtain and use patient/consumer and family feedback | 3 | Preparation | Medicaid agency, State behavioral health department | Educate beneficiaries about new benefits, solicit feedback | SUD treatment Providers, Beneficiaries, | One state and one federal comment period  States held multiple forums while drafting new benefits | Identify gaps in planned care continuum  [Appropriateness]  Raise awareness about planned services  [Acceptability, Adoption] | CMS required public comment periods on demonstration plans  States wanted to gain early buy-in for planned services | |
| Identify and prepare champions | 1 | Preparation | Medicaid agency innovation developers | Promote awareness and support for new Medicaid services | Champions in other state agencies, legislature | As needed | Obtain legislative approval for increased Medicaid budget  [Adoption] | Medicaid demonstrations must be budget neutral to CMS | |
| Access new funding | 3 | Preparation | Medicaid agency leadership | Education for legislature on Medicaid costs, new services | State legislature | Once, prior to submitting demonstration application to CMS | Obtain legislative approval for increased Medicaid budget  [Adoption] | Medicaid demonstrations must be budget neutral to CMS and financed by the state | |
| Promote network weaving | 3 | Implement-ation *(continued from Preparation)* | Medicaid agency innovation developers | Outreach to state Medicaid programs, non-governmental organizations, consultants  Education on SUD EBPs, strategies to implement EBPs | Medicaid benefit structure | Multiple calls, internet searchers | Identify EBPs to support SUD care continuum  [Appropriateness]  Identify lessons learned from implementation efforts in other states  [Feasibility] | Medicaid agencies were unfamiliar with EBPs | |
| Conduct educational meetings  Develop educational materials | 3 | Implement-ation | Medicaid agency | Education on EBPs, ASAM Criteria, documentation requirements | SUD treatment Providers | Varies by state, enough to train all Medicaid providers statewide | Promote fidelity to EBPs, ASAM Criteria  [Fidelity] | Providers were unfamiliar with ASAM Criteria | |
| Provide local technical assistance  Conduct educational outreach visits | 3 | Implement-ation | External vendors or Medicaid agency | Assess provider compliance to ASAM levels of care  Assign providers with an ASAM designation | SUD treatment Providers | Once per Medicaid provider | Promote fidelity to ASAM Criteria  [Fidelity] | Providers were unfamiliar with ASAM Criteria  Providers contracts an ASAM designation | |
| Alter incentive/ allowance structures | 3 | Implement-ation | Medicaid agency | Increase reimbursement rates for SUD services | SUD treatment Providers | Once when drafting new benefits | Expand provider network  [Adoption, Penetration] | Inadequate provider network | |
| Change credentialing and/or licensing standards | 2 | Implement-ation | Medicaid agency, State behavioral health department | Develop peer specialist support certification and training program | Peer support specialists | Continuous | Professionalize peer support specialist role  Expand provider network  [Adoption, Penetration] | Peer supports were a newly covered benefit under Medicaid | |
| Develop and distribute educational materials  Centralize technical assistance | 1 | Implement-ation | Medicaid agency | Create demonstration FAQ  Educate clinicians on EBPs | County Medicaid programs, clinicians, beneficiaries | Revisions throughout implementa-tion process | Promote awareness of demonstration  [Adoption]  Promote adoption of EBPs  [Adoption] | Medicaid is run on county-level, counties opted-in to demonstration | |
| Identify early adopters  Capture and share local knowledge | 1 | Implement-ation | Medicaid agency, CMS leadership | Showcase state and county implementation models | Other state Medicaid agencies  CA county Medicaid programs | Ongoing | Model implementation  [Adoption]  Share lessons learned  [Feasibility] | CA was the first state to implement a SUD 1115 waiver demonstration | |
| Stage implementation scale up  Centralize technical assistance | 1 | Implement-ation | Medicaid agency | Phased counties’ implementation of new benefits | County Medicaid programs | Ongoing | Focus resources on small groups of counties  Share lessons learned  [Feasibility, Adoptin] | Medicaid is run on county-level, counties opted-in to demonstration  Medicaid agency lacked staff to simultaneous implement benefits statewide | |
| Build a coalition  Develop resource sharing agreements | 1 | Implement-ation | Medicaid agency | Counties developed regional multi-county implementation models rather than single county models | County Medicaid programs, beneficiaries | Ongoing | Enhance feasibility  [Feasibility]  Adopt new services  [Adoption] | Smaller, under-resourced counties lack financial ability and/or provider network to implement new services | |
| Place innovation on FFS/ formularies | 1 | Implement-ation | Medicaid agency pharmacy benefit manager | Redesign Medicaid MOUD formulary | Medicaid MCOs | Once | Standardize and promote fidelity to MOUD benefits  [Fidelity] | Medicaid agency wanted MCOs to use a single formulary | |
| Alter incentive/ allowance structures | 1 | Implement-ation *(continued through Sustainment)* | State health department | Pay for providers to become DATA 2000 waivered | SUD treatment Providers | Ongoing as funding is available | Increase provider capacity to prescribe MOUD  [Adoption, Penetration] | Inadequate MOUD provider network | |
| Make billing easier | 1 | Implement-ation | Medicaid MCOs | Develop shared prior authorization, utilization management forms | SUD treatment Providers | Once | Standardize, streamline billing and service delivery processes  [Adoption, Penetration]  Improve fidelity to Medicaid benefits  [Fidelity] | Prevent providers from having to use different forms across 6 Medicaid MCOs | |
| Alter incentive/ allowance structures | 1 | Implement-ation | State behavioral health department | Allocate block grant funding SUD services | SUD treatment Providers | Initial months of implementa-tion | Reduce providers’ financial risk  Maintain provider network  [Adoption, Feasibility] | Support providers with bridge funding while reimbursement systems are established | |
| Use data experts  Work with educational institutions | 3 | Sustainment | Independent evaluator | Evaluate demonstra-tion’s impact on service access, quality | Medicaid agency | Annually, following completion of demonstration period | Measure changes in beneficiaries’ access to SUD services  [Sustainability] | CMS required task | |
| Alter patient/ consumer fees | 1 | Sustainment | Medicaid agency leadership, state legislature | Pass a state law banning providers from requesting cash payments from Medicaid beneficiaries | MOUD prescribers | Once | Improve fidelity to SUD benefits  [Fidelity] | Cash clinics lacked regulations, oversight, posed financial barriers to Medicaid beneficiaries’ accessing care | |
| Alter incentive/ allowance structures | 1 | Sustainment  *(continued from Implement-ation)* | State health department | Pay for providers to become waivered to prescribe buprenorphine | SUD treatment Providers | Ongoing as funding is available | Increase provider capacity to prescribe MOUD  [Adoption, Sustainability] | Inadequate MOUD provider network | |
| Develop and organize quality monitoring systems  Purposively reexamine the implementation | 3 | Sustainment | Medicaid agency | Assess provider network adequacy  Monitor service waitlists | Beneficiaries | Ongoing | Monitor adoption, fidelity to new benefits  [Sustainability] | Section 1115 waiver demonstrations are iterative processes | |
| *Abbreviations: American Society of Addiction Medicine, ASAM; Centers for Medicare and Medicaid Services, CMS; evidence-based practice, EBP; Institutions for Mental Disorders, IMD; N/A, not applicable; substance use disorder (SUD); United States, U.S.*  *Notes: *Implementation strategy names clusters were identified from the Expert Recommendations for Implementing Change project. Implementation strategy specification criteria were derived from Proctor et al. recommendations for specifying implementation strategies.*  *^+^Dose was difficult to specify for many strategies as stakeholders did not keep written records counting the number of times each strategy was used, especially if the strategy involved verbal communications with intermediaries. These details were also not recorded in policy documents.* | | | | | | | | |  |
